# The genetic counsellor role in the United Kingdom: Position on behalf of the Association of Genetic Nurses and Counsellors (AGNC), Endorsed by the Genetic Counsellor Registration Board (GCRB) and Academy for Healthcare Science (AHCS)

**DOI:** 10.1038/s41431-022-01212-9

**Published:** 2022-11-01

**Authors:** Anna Middleton, Nicola Taverner, Natalie Moreton, Roberta Rizzo, Catherine Houghton, Catherine Watt, Esther Horton, Sara Levene, Phil Leonard, Athalie Melville, Somya Ellis, Vishakha Tripathi, Christine Patch, Elaine Jenkins

**Affiliations:** 1 Engagement and Society, Wellcome Connecting Science, Wellcome Genome Campus, Hinxton, Cambridge, UK; 2 Kavli Centre for Ethics, Science, and the Public, Faculty of Education, University of Cambridge, Cambridge, UK; 3 School of Medicine, Cardiff University, Cardiff, UK; 4 All Wales Genetic Medicine Service, Cardiff, UK; 5 Manchester Centre for Genomic Medicine, Manchester University NHS Foundation Trust, Manchester, UK; 6 University Hospital Southampton NHS Foundation Trust, Southampton, UK; 7 Liverpool Centre for Genomic Medicine, Liverpool Women’s NHS Foundation Trust, Liverpool, UK; 8 Department of Clinical Genetics, West of Scotland Genetic Services, Glasgow, UK; 9 Nottingham University Hospitals, Nottingham, UK; 10 The Centre for Reproductive & Genetic Health, London, UK; 11 Birmingham Women’s NHS Foundation Trust, Birmingham, UK; 12 Wessex Clinical Genetics Service, Southampton, UK; 13 Guy’s and St Thomas’ Clinical Genetics Service, Guy’s Hospital, London, UK; 14 Head of Standards, Academy for Healthcare Science, London, UK

**Keywords:** Health care, Genetics

## Overview of the profession

Genetic counsellors are an internationally recognised group of highly skilled healthcare professionals with training and expertise in genomic medicine and counselling skills [1, 2]. The United Kingdom (UK) is a leader in developing the profession and has established the gold standard for genetic counselling emulated across Europe and elsewhere around the world [3, 4]. The delivery of genetic counselling in a clinical setting is underpinned by evidence, genetic counselling theory, uses established, validated outcome measures to evaluate success [5] and is highly valued by patients [6]. The number of genetic counsellors globally is approximately 7000, practicing in at least 28 countries [7]. In the UK, there are approximately 300 genetic counsellors, the vast majority practicing clinically in the publicly funded National Health Service.

The delivery of genomic testing had, until relatively recently, been the domain of clinical genetics services; however, such testing has now become ‘mainstream’, in that clinicians working across any discipline of healthcare can order certain genomic tests relevant to their patient. Such mainstream delivery of genomic medicine provides infinite opportunities for the profession of genetic counselling to support, mentor, train and offer clinical supervision to non-genetics colleagues who are utilising genomic technologies.

## Practicalities of the role

Genetic counsellors have expertise and skill that can be applied in every stage of the patient pathway within genomic medicine [4]. There is much cross over between the roles of the genetic counsellor and the clinical geneticist, and also variation in how these roles are defined within services across the UK. Clinical geneticists have expertise in diagnostic medicine and therapeutics whereas genetic counsellors have specific training and expertise in clinical genetics combined with counselling skills. Separate to the clinical geneticist role, there also remains a broad consistency of the genetic counsellor’s scope of practice.

Genetic counsellors are skilled and trained in: calculating genetic risk, explaining inheritance patterns, ordering genomic testing, interpreting variants, arranging medical and/or diagnostic testing as well as testing of relatives, predicting risks of genetic disease, referring patients for appropriate disease screening and handling all the consequent psychosocial and ethical issues raised for individuals and their families. They can also act as on-call specialists for urgent referrals to the clinical genetics service and triage referrals into the service. They are the main health professional group who ‘takes care of the family’, facilitating family communication, coping and adjustment as well as cascading information and arranging testing of at-risk relatives. A key role that distinguishes genetic counsellors is that of being patient advocates both within a clinical genetics setting but more so in their work across specialities where the best interest of the patient and/or the family may find itself in conflict with what is deemed ‘routine practice’.

In the UK, a clinical consultation usually takes, on average, 45 mins (with a range of 30–90 min based on the needs of the patient). Within a full day clinic, the number of patients seen will vary depending on the clinic type, patient concerns and complexity of the cases. However, on average, genetic counsellors may see between 7 and 10 patients per full day clinic. Genetic counsellors are skilled in delivering their consultations face-to-face, on the telephone or increasingly by virtual (video) clinics.

Classically, genetic counsellors work in tandem with their clinical geneticist and clinical laboratory scientist colleagues as part of a tertiary clinical genetics service (many of which have been renamed Genomic Medicine Services) in the UK. Continuation of this model remains vitally important; however, an uncoupling of these roles has already started for some genetic counsellors, who increasingly also work in other settings. ‘Uncoupling’ should not be read as ‘no longer working together’. It is important for genetic counsellors working outside of clinical genetics to be able to retain a link to this service for multidisciplinary team input, line management from consultant genetic counsellors and counselling supervision.

## Mainstream genetic counselling roles

Within ‘mainstream medicine’, i.e. outside specialised clinical genetic services, genetic counsellors can support and advise non-genetics clinicians to deliver genomic medicine. This includes supporting clinicians to order the most appropriate test for their patient and providing education so that colleagues understand the nuances of informed consent relevant to genomic testing. This ensures that resources are targeted appropriately, minimising the cost, delay and negative impact of inappropriate genomic testing. Genetic counsellors currently play a role in helping to discharge inappropriate recall of patients from disease screening programmes, by refining genetic risks and re-targeting screening to those most at risk of developing disease (for example, for cancer and cardiac services). They also sit as the genetics expert in multidisciplinary team meetings across hospital settings, offering advice on referral pathways, interpretation of genomics results, and patient management. Across the UK there are already genetic counsellors practicing autonomously within non-genetics specialisms, e.g. within the ophthalmology, cancer, endocrine, psychiatry, fertility, ENT and dermatology clinics.

There are also ‘mainstream’ genetic counsellors who, whilst attached to their local clinical genetics service, work directly with their non-specialist colleagues across a hospital who are delivering genomic medicine services that do not need the specialist input of clinical genetics. Genetic counsellors have been welcomed by many disciplines outside of clinical genetics, and often act as the link between services, ensuring and supporting the competent delivery of genomic medicine by non-genetics healthcare professionals. Models of genetic counselling delivery are evolving, and this is a reflection of the adaptability and transferability of the genetic counselling skill set.

In many cases, results of genomic tests done within mainstream settings may have implications for disciplines outside of the medical specialty ordering the test. For example, a variant predisposing a patient to inherited heart disease may be identified as an ‘additional looked for finding’ in a patient in a cancer clinic; or an adult patient in a lipid clinic may have a variant identified that predicts a risk of disease to offspring, and so reproductive options need to be explored. In these situations, genetic counsellors working in mainstream settings can facilitate onward referrals or co-ordinate/manage the cases themselves, for example for cascade genetic testing of relatives, prenatal diagnosis or preimplantation genetic diagnosis (PGD).

In the majority of cases, the results and information obtained from genomic analysis have implications not only for the index case but their relatives. For every new genetic diagnosis made in a non-genetics healthcare setting, the clinician who orders the test and delivers the results, should have a conversation about the impact of these for relatives. Given that non-genetics clinicians would not routinely care for at-risk, healthy relatives, the future management of such ‘patients in waiting’ can be managed via a genetic counsellor.

In addition to established clinical practice, the genetic counsellor role has also evolved and now genetic counsellors practice as leaders in research, policy, education (both internal and external to the profession), private practice, service management and professional regulation.

## Genetic counsellor regulation

The genetic counselling profession established a professional accreditation process overseen by the Genetic Counselling Registration Board (GCRB), with recognised routes of entry into the profession and applicants required to deliver a Masters-level portfolio of evidence to demonstrate professional competence. In 2019 the GCRB transitioned under the auspices of the Academy for Healthcare Science (AHCS) and is now an integral part of the Academy’s regulatory function. The AHCS maintains the UK register of Healthcare Science practitioners which is accredited by the Professional Standards Authority for Health and Social Care (PSA) on an annual basis. Genetic counsellors are currently assessed by the GCRB for inclusion on the AHCS accredited register and are expected to meet the professional standards of competency and conduct as set by the GCRB and approved by the AHCS. Any concerns or complaints made about a GCRB registrant will be raised with the AHCS, which will initiate and manage a Fitness to Practise investigation if it is found to be necessary. The expectation of employers is that, in order to be able to practice, genetic counsellors must be registered with a recognised registration body which, for most genetic counsellors in the UK means the AHCS.

The NHS Scientist Training Programme now has a genomic counselling branch leading to statutory registration with the Health and Care Professions Council (HCPC). Here, the legally protected title that genetic counsellors will use is ‘Clinical Scientist’, and, if they so choose, they can suffix this with genetic or genomic counsellor. The AHCS offers an equivalence route for those genetic counsellors who have not had the opportunity to undertake the Scientist Training Programme. HCPC registered genomic counsellors may also apply for AHCS registration through the GCRB if they wish and some have chosen to do so to enhance collective belonging within the profession.

The AHCS promotes genetic counsellor registration to ensure protection for the patients and public they serve.

## Position within mainstream genomic medicine service delivery

Historically, the profession of genetic counselling emerged out of nursing practice. Indeed, in the UK, alongside the Masters training in genetic counselling, there is a specialist nursing route into the profession as well. With the advent of the new Genomic Medicine Alliances in England (GMA) (nursing, medicine and pharmacy), it is likely that the profession of genetic counselling can be aligned with the Nursing GMA, which will be different from medically trained clinical geneticists colleagues who could naturally align with the Medicine GMA.

Classic models of line-management are evolving, such that whilst many genetic counsellors will remain within the specialised clinical genetics services, they may also be commissioned via other funding routes and thus work and are line-managed independently of the traditional clinical geneticist/clinical laboratory scientist/genetic counselling model of service delivery. Commissioning models need to consider the allocation of resources specifically to genetic counsellors working separately to clinical genetics services.

As a professional body, the AGNC supports the evolution of the genetic counselling role so that independently practicing genetic counsellors can function, allied to, but working within, services external to the traditional clinical genetics model of care. Genetic counsellors need to be accountable for their own practice and to their own professional regulator as opposed to needing to be ‘responsible to’ clinical geneticists or another mainstream medical colleague. Nowadays, it is more usual for a genetic counsellor to be line-managed and held accountable to a consultant genetic counsellor, rather than a consultant clinical geneticist. For genetic counsellors working in mainstream settings, they may wish to retain formal links with their consultant genetic counsellor colleagues for line-management, mentorship and counselling supervision.

## Training others

As the genetic counsellor is highly skilled in the adaptation of complex information for families, it follows that they have an essential role to play in the delivery of educational materials, resources and teaching for healthcare professionals. Genetic counsellors have long held responsibilities for providing genetic risk estimation training to non-genetic healthcare providers such as breast care nurses based in NHS breast care units or foetal management training to specialist midwives and registrars training in foetal medicine. Training has also extended to GPs.

## Workforce planning

With the implementation of new genomic medicine services across the NHS, hundreds of thousands of new patients will routinely undergo genomic testing as part of their diagnostic pathway. Many genomic results will not only have implications for the patient, but also for their biological relatives too. ‘Care of the family’ and testing of relatives will necessitate a referral of the patient, post result, into a clinical service that can cascade out testing within a family. Genetic counsellors are capable of picking up these referrals and either delivering the genetic counselling themselves or mentoring/line managing other health professionals to do this (e.g. specialist nurses, doctors). Workforce planning and the need for more skilled staff, including genetic counsellors, is recognised as an issue that needs addressing both in the UK and internationally.

Urgent modelling is needed to explore how the role of the genetic counsellor can evolve to meet the demands of genomic medicine delivered outside of traditional clinical genetics services. Genetic counsellors are an agile profession capable of playing a pivotal role in supporting the delivery of genomic healthcare both within existing pathways but also within new service delivery.
